# CT Enterography score: a potential predictor for severity assessment of active ulcerative colitis

**DOI:** 10.1186/s12876-018-0890-z

**Published:** 2018-11-09

**Authors:** Yingmei Jia, Chang Li, Xiaoyan Yang, Zhi Dong, Kun Huang, Yanji Luo, Xuehua Li, Canhui Sun, Shi-Ting Feng, Zi-Ping Li

**Affiliations:** 1 Department of Radiology, The First Affiliated Hospital, Sun Yat-Sen University, 58th, The Second Zhongshan Road, Guangzhou, 510080 Guangdong China; 2 Department of Radiology, Shenzhen Traditional Chinese Medicine Hospital, Shenzhen, 518000 China

**Keywords:** Ulcerative colitis, Disease activity, Multi-slice computed tomography, Computed tomography enterography, Modified Mayo score

## Abstract

**Background:**

Evaluate the possibility of CT enterography (CTE) score system as a predictor in assessing active ulcerative colitis (UC) severity.

**Methods:**

Forty-six patients with active UC with CTE and colonoscopy were enrolled. Based on modified Mayo score, patients were divided into three groups: mild (*n* = 10), moderate (*n* = 17) and severe (*n* = 19). A cumulative CTE score was calculated in each patient and its correlation with modified Mayo score was analyzed. The optimal cutoff values of CTE score were determined by receiver operating characteristic (ROC) curves analysis.

**Results:**

Significant between-group differences were observed in CTE spectrums of mucosal bubbles, mural stratification, loss of haustration, enlarged mesenteric lymph nodes and engorged mesenteric vessels (*P* < 0.05). The cumulative CTE scores were significant difference between three groups (CTE score:4.9 ± 2.3, 7.6 ± 2.6, and 10.9 ± 2.0, respectively, *P* < 0.01). The cumulative CTE score showed a positive correlation with modified Mayo score (*r* = 0.835, *P* < 0.05). The optimal cut-off value for CTE score predicting moderate and severe UC was 9.5 (area under the curve [AUC]:0.847, sensitivity:78.9%, specificity:82.4%).

**Conclusion:**

Disease severity assessment by CTE score demonstrates strong positive correlation with severity established modified Mayo score. CTE score system maybe a potential predictor for active UC severity assessment.

## Background

Ulcerative colitis (UC) is a chronic non-specific inflammatory bowel disease (IBD) characterized by diffuse inflammation of bowel mucosa and a relapsing disease course. UC mainly affects the rectum and sigmoid colon, but may involve the entire colon and terminal ileum [1–3]. As UC is a chronic condition, management of these patients requires prolonged treatment and regular follow-up throughout the course of the disease. Accurate evaluation of UC is a crucial component of therapeutic decision-making [4, 5]. However, owing to the non-specific symptoms, assessment of UC typically requires a combination of colonoscopic, histological and radiological examinations in addition to clinical examination [6].

Modified Mayo score is used frequently for assessment of UC activity [7], mainly depending on patient’s symptoms and colonoscopy. Colonoscopy affords direct visualization of the colonic mucosa and has been the preferred method for defining the extent and site of inflammation, and to obtain biopsy specimen [8]. However, colonoscopy does not provide information on extra-intestinal manifestations and complications of UC. Moreover, in severe cases of UC, colonoscopy is contraindicated due to the risk of perforation or exacerbation of disease activity [9]. At present, computed tomography enterography (CTE), with its high contrast resolution and rapid images capability, allows evaluation of intramural and extra-intestinal involvement of UC and complications such as fistula, abdominal abscess or cellulitis. CTE is now widely used to diagnose and monitor inflammatory bowel disease (IBD), including UC [10, 11].

Establishment of a quick and accurate method to predict the severity of UC is an important goal for improving management of patients in future. Depending on the symptom, endoscopy and histology findings to predict the severity of UC is quite complex and time-consuming. Several studies have suggested the utility of CTE for assessment of severity of UC and a positive association of CTE findings with clinical and colonoscopic findings has been demonstrated [11]. Thus, identification of a CTE score system to assess and predict the severity of active UC is possible. However, to the best of our knowledge, no previous studies have investigated the utility of CTE score in predicting the severity of UC.

In this retrospective study, we assessed the individual CTE features in different severity of active UC, established a new CTE score system for UC, and analyzed the correlation between cumulative CTE score and modified Mayo score, so as to investigate the possibility of CTE score system as a predictor in assessing the severity of active UC.

## Materials and methods

### Patients

This is a retrospective study conducted in accordance with ethical guidelines for human research and was compliant with the Health Insurance Portability and Accountability Act (HIPAA). The study received ethical committee approval. The requirement for informed consent was waived off.

This study included 46 consecutive patients (29 men and 17 women) with a mean age (±SD) of 40.9 ± 17.2 years (age range: 19–77) at the First Affiliated Hospital, Sun Yat-Sen University between January 2010 and December 2015. The diagnosis of active UC was based on colonoscopic, clinical and histopathological examination. All patients had undergone CTE within 7 days of colonoscopy with biopsy.

The main symptoms included abdominal pain, fever, nausea and vomiting, intestinal bleeding and diarrhea. The duration of the disease course ranged from 1 month to 18 years.

### Modified Mayo score

Reference the European consensus on diagnosis and management of UC reported by Dignass [7], classification of active UC was evaluated by modified Mayo scoring system (Table 1).

**Table 1:** Components of the modified Mayo score [20]

Stool frequency	
0: Normal	
1: 1–2 stools/day more than normal	
2: 3–4 stools/day more than normal	
3: > 4 stools/day more than normal	
Rectal bleeding^a^	
0: None	
1: Visible blood with stool less than half the time	
2: Visible blood with stool half of the time or more	
3: Passing of blood alone	
Mucosal appearance at endoscopy^b^	
0: Normal or inactive disease	
1: Mild disease (erythema, decreased vascular pattern, mild mucosal friability	
2: Moderate disease (marked erythema, absent vascular pattern, friability, erosions)	
3: Severe disease (spontaneous bleeding, ulceration)	
Physician rating of disease activity	
0: Normal	
1: Mild	
2: Moderate	
3: Severe	

^a^A score of 3 for bleeding required patients to have at least 50% of bowel motions accompanied by visible blood, and at least one bowel motion with blood alone

^b^Mucosal appearance at endoscopy is not included in the Partial Mayo Score < 2, remission; 3–5, mild active; 6–10, moderately active; 11–12, severe active

### CTE technique

Patients were asked to fast for more than 12 h before the CT scan. Cleaning enema was performed on the night before the examination. Prior to scan, patients ingested a total of 1600~ 2000 mL of 2.5% mannitol solution in 400–500 mL aliquots every 15 min, and administered 20 mg of anisodamine intramuscularly. For achievement of adequate entire colon distension, colon retention enema with 300~ 500 mL of 2.5% mannitol was performed before CT scan [12, 13]. Patients were scanned using Toshiba Aquilion 64-detector row CT from diaphragmatic top to ischial tuberosity. Three-phases were obtained; before injection of contrast (non-contrast), and 23–25 s, and 50–60 s after intravenous administration of 1.5 mL/Kg iopromide (ultravist 300, Schering, Berlin, Germany) injected at a rate of 3–4 mL/s using a power injector. CT scan parameters were 120 kv, 200–250 mAs, collimation 64 × 0.5 mm, section thickness and interval of 2 mm.

### CTE image analysis

All data were reviewed on the workstation (Vitrea version 3.7). Each CTE study was evaluated by two trained abdominal radiologists who were blinded to the clinical and colonoscopic findings. Discrepancy, if any, was resolved by consensus.

With bowel well distended, bowel wall thickening was defined as > 4 mm [5]. Based on the Montreal classification [14], the extent of the disease was classified as E1, E2 and E3. E1 defined a proctitis with limited lesions until rectosigmoid junction, E2 defined a left-sided colitis with lesions under the splenic flexure and E3 defined an extensive colitis above the splenic flexure. Mural stratification was defined as circular intestinal wall and submucosal widening with decreased attenuation. Compared with the adjacent normal bowel, the features of mural hyperenhancement and mesenteric hyperemia were presented as increased mural density and engorgement of mesenteric vessels after enhancement. Perirectal stranding was depicted as a slightly increased attenuation (10-20HU) compared with normal fat as result of edema and inflammatory cell infiltrates [15]. Mesenteric lymph nodes with short axis > 5 mm were defined as enlarged. Mucosal bubbles present as round small bubbles in interrupted mucosal layer of intestinal wall. Intestinal pseudopolyp formed by colonic mucosal hyperplasia were shown as hummocky or nodules sample protrusions swelled to lumen. Luminal narrowing was defined by lack of full expansion of the bowel. Loss of haustration was also a feature of CTE images.

Once one of the active UC CTE features in any segment, a point was added. A cumulative CTE severity score (0–15 points) was calculated as the sum of all individual criteria scores. The cumulative CTE scores are shown in Table 2.

**Table 2:** Cumulative CT scores

CTE images	Scores
Extended range of UC	
Normal	0
E1	1
E2	2
E3	3
Bowel wall thickening	
< 3 mm	0
4–6 mm	1
7–9 mm	2
> 10 mm	3
Mural stratification	1
Mural hyperenhancement	1
Mesenteric hyperemia	1
Perirectal stranding	1
Lymph node enlargement	1
Mucosal bubbles	1
Luminal narrowing	1
loss of haustration	1
Intestinal pseudopolyp	1

### Statistical analysis

All data were analyzed by SPSS 17.0 software. Numerical data were expressed as mean ± SD and analyzed using R*C table method of Chi-squared test. After the difference is considered statistically significant among groups, partitions method of chi-square test were used to analyze. One-way Analysis of Variance (ANOVA) was performed for analysis of cumulative CTE score in 3 groups with the two-two comparisons. Correlation of cumulative CTE scores with the Mayo score was assessed using Spearman correlation (r). A *P* value of < 0.05 was considered statistically significant. Receiver-operating characteristic (ROC) curves were used to determine the optimal cut-off points of the CTE score for predicting severity of active UC.

## Results

### Modified Mayo score

According to the modified Mayo score, 46 patients were graded as mild (10), moderate (17) and severe (19) groups.

### CTE

According to the standard of study of Wold PB et al. [16], adequate luminal distention was defined as separation of the colon lumen by enteric contrast material without collapse. All patients (*n* = 46) had adequate distension of the entire colon.

In the total of 46 patients, involvement of the rectum was 2 cases (E1), involvement of a proportion of the colorectum distal to the splenic flexure was 23 cases (E2), and involvement extends proximal to the splenic flexure was 21 cases (E3). Overall, the consistency of CTE in assessment the extent of UC with endoscopy was 80.4% (80.0%, 82.3% and 79.0% for mild, moderate and severe group, respectively).

**Fig. 1:**
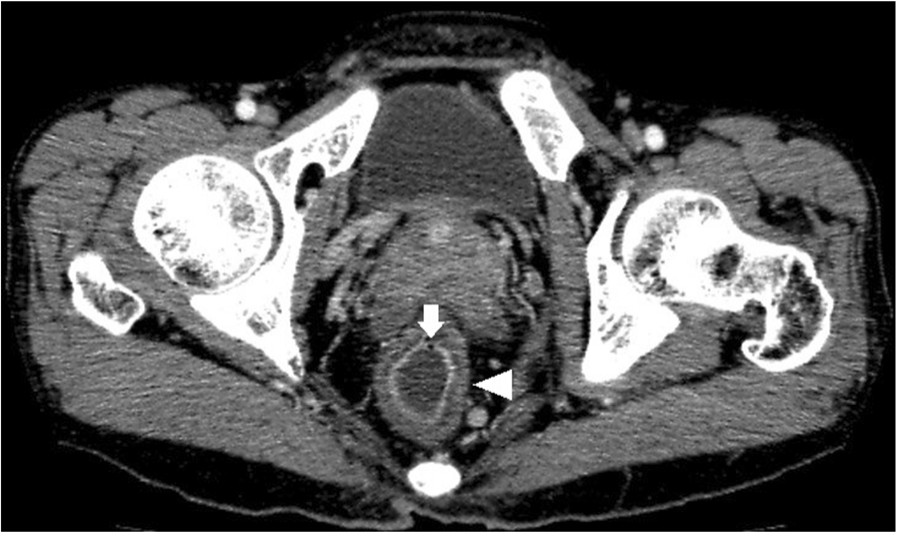
Axial CTE of UC showed uniform mural thickening and stratification in rectum (arrowheads). Mucosal hyperenhancement and mucosal bubbles (arrows) sign were detected

**Fig. 2:**
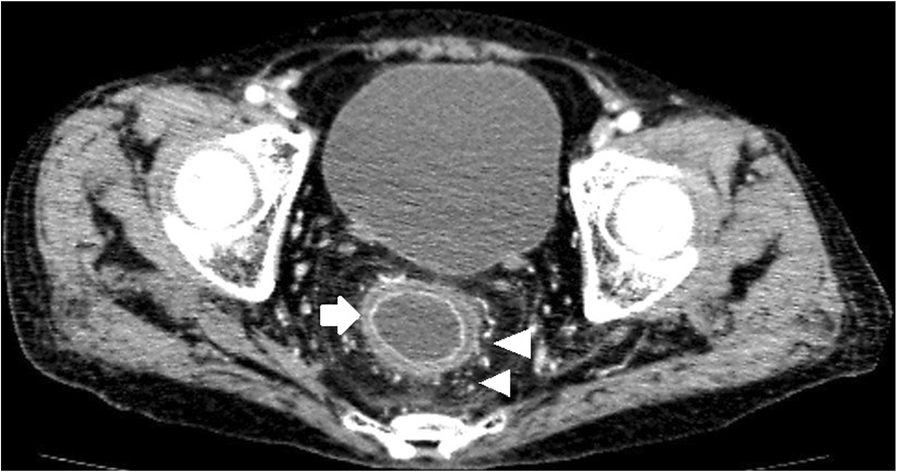
Axial CTE of UC revealed bowel wall thickening, mural stratification and mural hyperenhancement (arrows) in rectum. Perirectal stranding was observed (arrowheads) displaying increased attenuation in perirectal fat

**Fig. 3:**
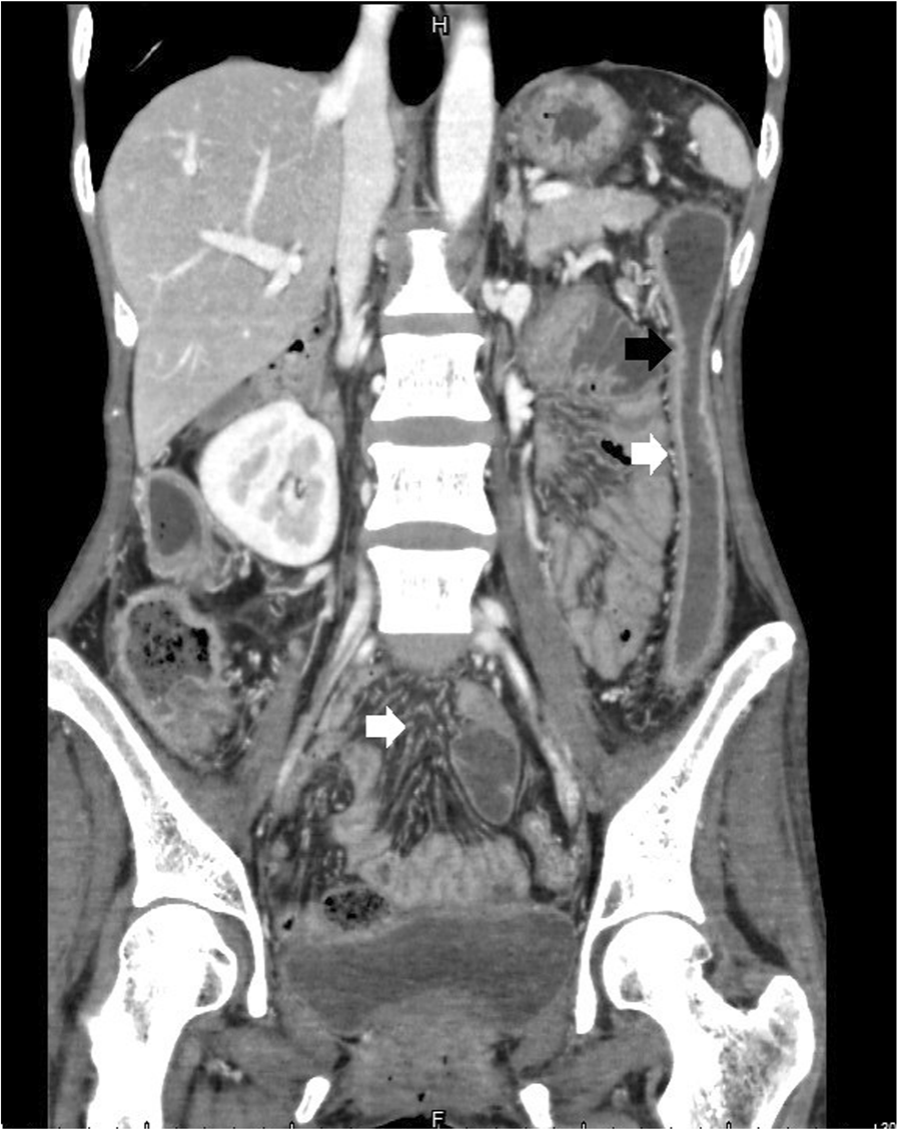
Coronal CTE of UC showed loss of haustration, lumenal narrowing, mucosal hyperenhancement (black arrows) and engorgement of mesenteric vessels (white arrows)

**Fig. 4:**
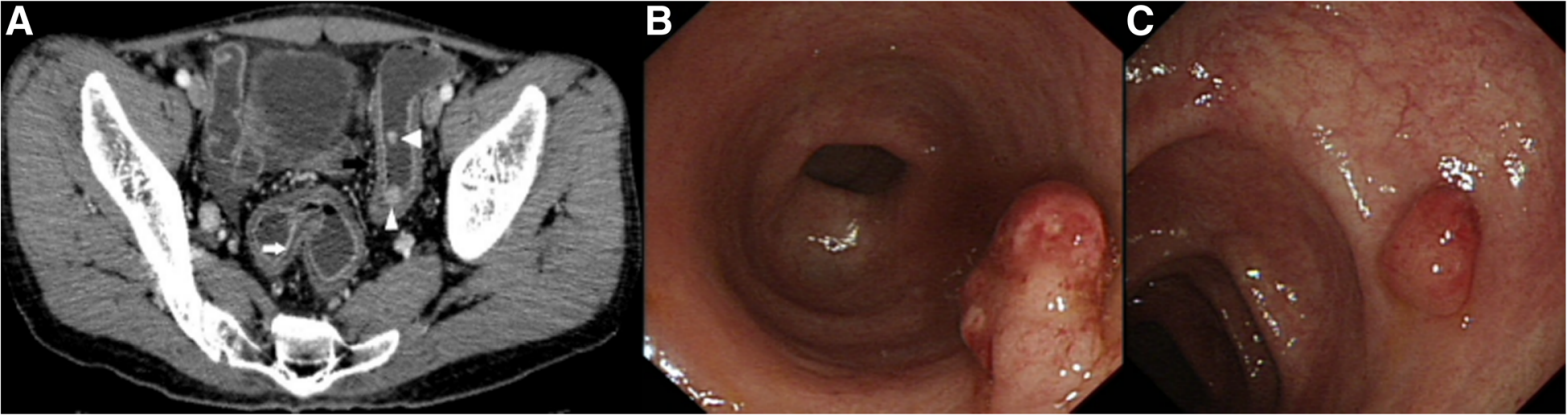
Axial (**a**) and CTE of UC demonstrated two enhancing mucosal nodules in sigmoid colon (white arrowheads). Colonoscopy-guided biopsy (**b** and **c**) in sigmoid colon confirmed them both to be pseudopolyps

Of the total 46 cases, bowel wall thickening was seen in 43 cases (Figs. 1, 2, 3, 4). Mural hyperenhancement was observed in 45 cases (Figs. 1, 2, 3, 4). Mural stratification was present in 21 cases (Figs. 1, 2, 4). Mucosal bubbles were present in 30 cases (Fig. 1). Loss of haustration was identified in 28 cases (Fig. 3). Mesenteric hyperemia was present in 23 cases (Figs. 2, 3, 4). Perirectal stranding was seen in 14 cases (Fig. 2). Lymph node enlargement was present in 19 cases. Intestinal pseudopolyps were identified in five patients (Fig. 4). Luminal narrowing was seen in 12 cases (Fig. 3).

### Statistical result

Among the individual CTE features, a significant difference in the spectrum of mucosal bubbles was observed between the mild and moderate groups (*P* = 0.003). A significant difference with respect to mural stratification, loss of haustration and enlarged mesenteric lymph nodes was observed between the moderate and severe groups (*P* = 0.019, *P* < 0.001, *P* = 0.003). Further, as significant difference was observed with respect to mucosal bubbles, loss of haustration, engorged mesenteric vessels and enlarged mesenteric lymph nodes between the mild and severe groups (*P* = 0.016, *P* < 0.001, *P* = 0.002, *P* = 0.002). There was no significant difference in the spectrum of bowel wall thickening, mural hyperenhancement, luminal narrowing and perirectal stranding between the 3 groups (*P* > 0.05). The spectrum of CTE features in 3 groups is shown in Tables 3 and 4**.**

**Table 3:** Spectrum of CTE features in 3 groups

CTE features	Groups			*P*
	Mild (*n* = 10)	Moderate (*n* = 17)	Severe (*n* = 19)	
Extended range				0.309
E1	1	1	0	
E2	7	8	8	
E3	2	8	11	
Bowel wall thickening	9	15	19	0.318
Mural hyperenhancement	8	15	19	0.164
Mural stratification	3	5	13	0.034
Mucosal bubbles	2	14	14	0.003
loss of haustration	2	7	19	< 0.001
Mesenteric hyperemia	1	8	14	0.005
perirectal standing	1	6	7	0.282
Lymph nodes enlargement	1	4	14	0.001
Intestinal pseudopolyp	0	2	3	0.426
Luminal narrowing	1	4	7	> 0.05

**Table 4:** *P* values for between-group differences on ANOVA

groups	CTE features				
	Mural stratification	Mucosal bubbles	loss of haustration	Mesenteric hyperemia	Lymph node enlargement
Mild-Moderate	1.000	0.003	0.406	0.091	0.621
Moderate-Severe	0.019	0.695	< 0.001	0.102	0.003
Mild-Severe	0.064	0.016	< 0.001	0.002	0.002

**Fig. 5:**
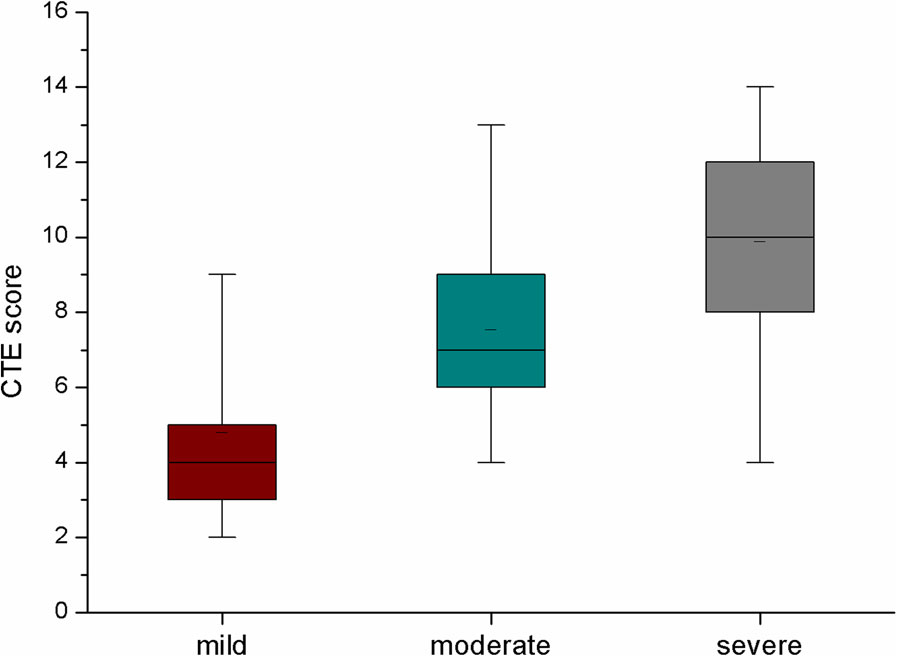
Distribution of the cumulative CTE score

**Fig. 6:**
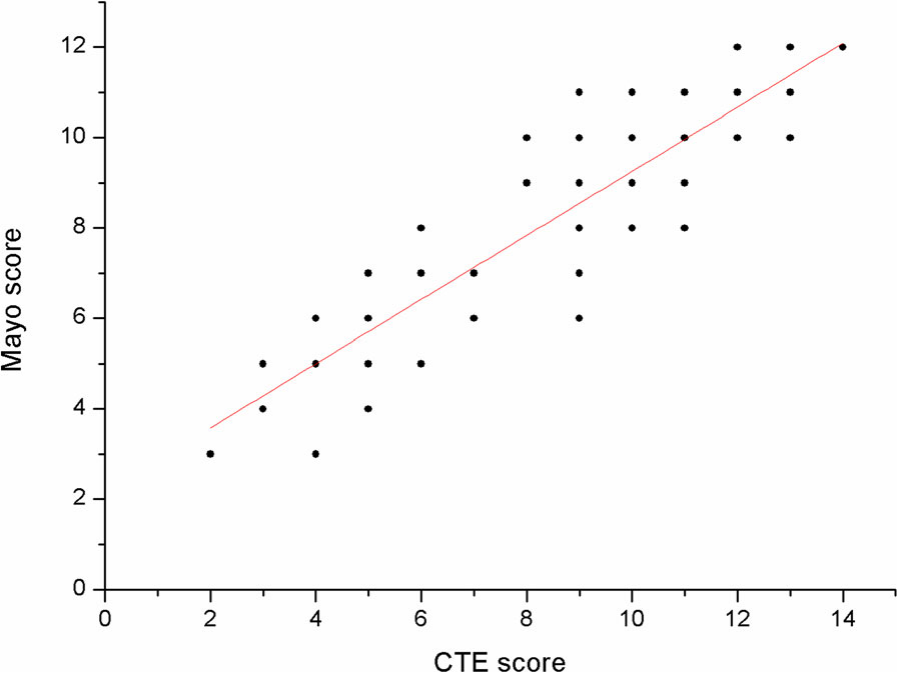
Correlation between CTE score and Mayo score in patients with ulcerative colitis *Note: Because some of the cases have exactly the same CTE score and Mayo score, there is some overlap of the scatter plots

The cumulative CTE scores for mild, moderate and severe groups were 4.9 ± 2.3, 7.6 ± 2.6, and 10.9 ± 2.0 (Fig. 5) with a statistically significant difference among the three groups (*P* < 0.01). On correlation analysis, a positive linear correlation between the cumulative CTE score and modified Mayo score was observed (*P* < 0.05, *r* = 0.835) (Fig. 6).

**Fig. 7:**
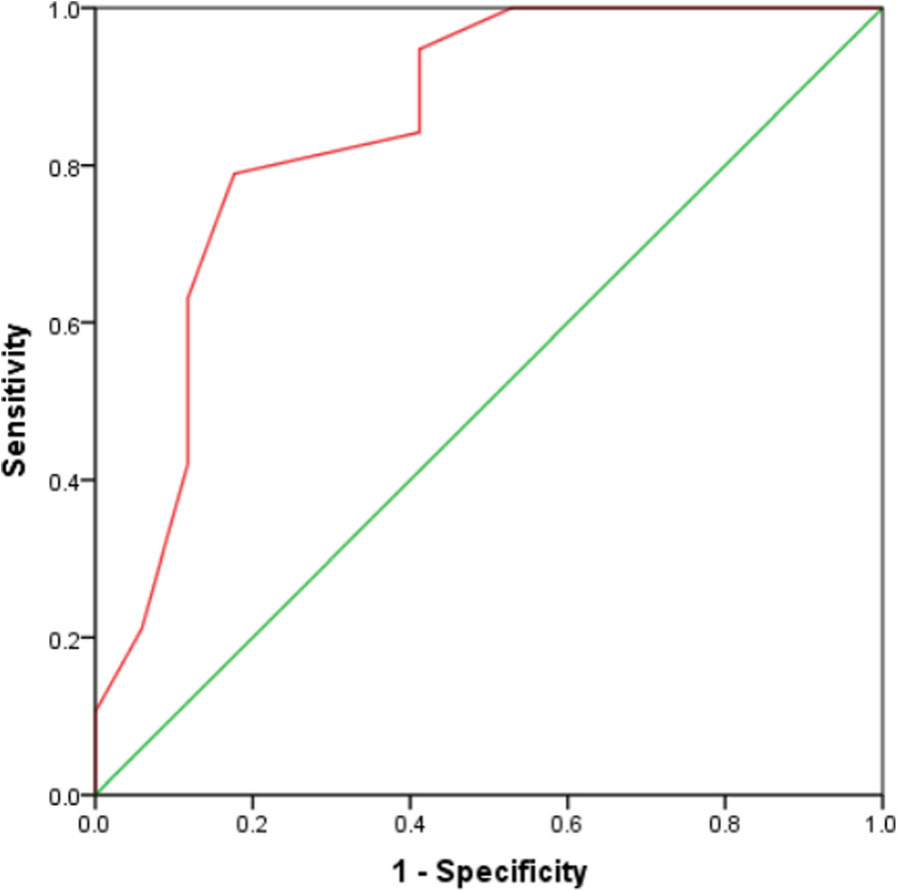
Area under the curve (AUC) of the receiver-operating characteristics (ROC) for differentiation of moderate and severe group of CTE score

The optimal CTE score cut-off value for predicting moderate and severe UC was 9.5 with an area under the ROC curve of 0.847 (Fig. 7). The sensitivity and specificity were 78.9% and 82.4%, respectively. There was no optimal cut-off value for predicting mild and moderate UC due to the low AUC (0.280).

## Discussion

The symptoms of active UC include persistent or recurrent episodes of diarrhea, intestinal bleeding with abdominal pain, tenesmus and different levels of systemic symptoms [2]. Repeated bouts of inflammation lead to chronic active UC, and increase the grade of activity [11]. Based on the extent of involvement and degree of inflammatory activity, the active inflammatory severity of UC was divided into mild, moderate and severe disease.

In patients with UC, an exhaustive evaluation of disease extent and activity is crucial for therapeutic decision-making [17–19]. Therapeutic planning and follow-up is related to disease severity [10]. Modified Mayo score comprised of four categories (stool frequency, rectal bleeding, endoscopic appearance and physician assessment) is common used to evaluate severity of active UC [2, 20]. Nevertheless, these are indirect indices, mainly relying on self-assessment of symptoms by the patient [21]. Moreover, endoscopy can not provide information on bowel wall, extraintestinal manifestations and complications of UC [22].

CTE with increased speed and resolution, allows for comprehensive assessment of the bowel wall and extra-intestinal manifestations and becomes a useful compliment to endoscopy [23–25]. A few studies have focused on the accuracy of CTE for the detection of UC. Johnson et al. [26] reported that an overall sensitivity of 74% for the detection of IBD (either Crohn or UC), and sensitivity was 93% for the detection of moderate and severe disease in well-distended colons and specificity was 91%. Andersen et al. [27] reported a moderate correlation of the loss of haustration, rigid bowel wall, and bowel thickness with severity of UC. Our study and previous studies showed that CTE was highly correlated with colonoscopic findings in assessment the extent of UC. Consequently, CTE is an ideal method and has great potential in evaluation for UC even at earlier occasion.

CTE spectrum are correlated closely with pathological findings and has the ability to reflect the pathological changes of UC. Pathologically, the early stages of UC are characterized by mucosal hyperplasia, increased mucosal vascularity, congestion and edema, which present as mucosal hyperenhancement and bowel wall thickening on CTE. With progression of disease course, the disease is characterized by multiple mucosal erosions or ulcers, which may manifest as mucosal bubbles in interrupted mucous. With further development of UC, the disease is characterized by edema, congestion, and inflammatory cells infiltration of submucosa, and hyperplasia and chronic fatty deposits of muscular layer. On CT, these changes appear as mural stratification and loss of haustration. In addition, luminal narrowing due to hyperplasia and fibrosis of muscularis mucosa occurs along with enlarged mesenteric lymph nodes and engorged mesenteric vessels due to chronic inflammation. Also, perirectal stranding is hallmark of chronic disease [11, 27, 28]. These pathological characteristics allow an objective assessment which reflects the severity of UC.

In our study, the spectrum of CTE findings in the three groups was difference. Bowel wall thickening and mucosal hyperenhancement were the essential characters and almost observed in all the cases. However, mucosal bubbles were more frequently observed in the moderate group as compared to that in the mild group. Mural stratification, loss of haustration and enlarged mesenteric lymph nodes in the severe group were significantly higher than that in the moderate group. Compared with the mild group, patients in the severe group were more likely to show mucosal bubbles, loss of haustration, engorged mesenteric vessels and enlarged mesenteric lymph nodes. Thus, increasing severity of UC demonstrated various CTE findings, reflecting the corresponding severity of intestinal inflammation. In mild UC, the intestinal inflammatory was mainly located in the mucosa and submucosa. In more severe disease, the inflammation spread from mucosa and submucosa to the whole intestinal wall, including ulceration and edema [27]. Small and tiny ulcerations are hard to detect by CTE. Mucosal bubbles represents obvious ulcer indicating more serious inflammatory activity. Mural stratification and loss of haustration indicated the UC involving the whole intestinal wall suggesting the severe disease. However, no statistically significant difference was found between the three groups with respect to luminal narrowing, perirectal stranding and pseudopolyp. Because these three signs may be more related to disease duration and individual differences rather than the disease severity [29].

Due to the correlation between the extent of UC and the clinical manifestations, the wider the extent of UC is, the worse are the symptoms. Due to this, the extent of UC was added to the CTE score system. A synthesis of all CTE features provides for a quantitative score system to evaluate the severity of UC. Our results showed a statistically significant difference in the CTE scores between the three groups. Moreover, the CTE score strongly correlated with modified Mayo score (*r* = 0.835). With aggravation of the disease course, CTE score increased significantly. Patel et al.*..* [11], reported a weak correlation between the composite CT severity score and clinical assessment (*r* = 0.45), while bowel thickening, mucosal hyperenhancement, and mural stratification each individually showed a moderately positive association with clinical severity (*r* = 0.58; 0.57; 0.68). One possible reason is that our sample size is relatively large, and the modified Mayo scoring system (including colonoscopy) was used to evaluate active UC. Moreover, compared with the study of Patel et al [11], we developed a more comprehensive CTE score system in which CTE features correlated with disease severity as reflected in the extent of UC, mucosal bubbles, luminal narrowing and loss of haustration. Finally, we used plenty of low-density contrast media to expand the colon and the small intestine. The intraintestinal CT characteristics of UC such as mucosal bubbles, mural stratification, mural hyperenhancement may be displayed clearly with use of such agents, as opposed to high-density contrast media.

Accurate prediction of severity of active UC using a convenient method is necessary for management. Current methods (depending on the symptom, endoscopy and histology findings) to predict the severity of UC have some limitations. CTE provides more valuable information for judgment of active UC severity and maybe predict the severity. To our best knowledge, there are few studies using CTE score to predict severity of UC. In our study, based on ROC analysis, the CTE score system has the potential ability of predicting severity of UC. Our study showed CTE score was better for predicting moderate and severe disease of active UC with a CTE score cut-off value of 9.5 with high specificity. But there was no optimal cut-off value for predicting mild and moderate UC. This is probably due to the selection bias for the relative small number of mild UC. Another possible reason is there are more overlapping CTE findings between mild and moderate UC. Moreover, the CTE features are easier to identify with increasing inflammation degree. Even so, patients with UC whose CTE score more than 9.5 should be considered as having moderate or severe inflammatory severity. It is strongly recommended that such patients should be hospitalized.

Consequently, CTE enabled a comprehensively assessment of UC, helped determine the optimal treatment strategy and, to some extent, made up for the limitations of the conventional CT, as development of new signs on CTE, such as mucosal bubbles and loss of haustration showed a correlation with pathological changes of UC. These advantages are of clinic relevance in the diagnosis and classification of UC. Thus, CTE can be a reliable examination method that helps in systematic evaluation of the severity of UC.

There are some limitations in our study. Firstly, the score given to each characteristic should ideally be derived based on regression analysis. However, for our study, the sample size is relative small. Most of the cases are severe groups (41.3%, 19/46) and selection bias can not be avoided. Thus, we did not use regression analysis in building CTE score system. Secondly, this CTE score system cannot be reliably used as a predictor of the outcomes of UC; however, it remains a very interesting subject to investigate. These need a further study enrolling a larger number of patients from multiple centers.

## Conclusions

CTE enables assessment of extraintestinal manifestations and complications of UC. And, CTE score system provides a quantitative basis for accurate assessment of the severity of UC. Thus, CTE is a valuable supplement to the traditional methods such as colonoscopy and clinical assessment. It can be used as a potential predictor of severity assessment of active UC, help determine therapeutic strategy, and predict prognosis.
